# Impact of the Harm Review Service for Patients Awaiting Elective Hip and Knee Surgeries for More Than 52 Weeks

**DOI:** 10.7759/cureus.23805

**Published:** 2022-04-04

**Authors:** Anand Divekar, Omkaar Divekar, Devaraj M Navaratnam, Raj Shrivastava

**Affiliations:** 1 Trauma and Orthopaedics, William Harvey Hospital, Ashford, GBR; 2 Trauma and Orthopaedics, St. George’s University, London, GBR

**Keywords:** arthritis, mental wellbeing, delay in surgery, elective orthopaedic surgery, hip and knee replacement

## Abstract

Background

The coronavirus disease 2019 (COVID-19) pandemic has affected medical practice worldwide. In the UK, elective operative lists had to be postponed to accommodate the increase in hospital admissions. Within our local trauma and orthopaedic department, a harm review clinic was developed for these postponed elective cases. The purpose of this clinic was to evaluate the impact and outcomes of the delay in elective hip and knee procedures.

Methodology

The elective list database of William Harvey Hospital, Kent, from April to December 2020 was retrospectively analysed. Inclusion criteria included all lower limb primary arthroplasty, elective lower limb revision surgery, and other hip and knee procedure patients waiting more than 52 weeks for surgery. All patients had telephone consultations averaging 10 minutes. Data included patients’ symptoms, fresh investigations, changes in treatment plans, mental health status, and value of consultation were assessed and recorded.

Results

A total of 242 patients from eight lower limb consultants were analysed. Patients with hip pathology accounted for 39.2% (95 patients) versus knee pathology accounting for 60.7% (147 patients). In total, 13 (5.37%) patients reported improvement in their physical symptoms, whereas 46 (19%) felt their symptoms worsen. Overall, 26 (10.7%) patients had a change in their treatment plan following the consultation. In total, 18 (7.4%) patients required further face-to-face follow-up following the telephone consultation There were no patients who had significant physical or mental harm.

Conclusions

The COVID-19 pandemic has brought changes in how we practice medicine. The harm review service has been a valuable service to both patients and the orthopaedic department. This harms review clinic was able to identify changes in treatment plans for patients. A small percentage of patients required face-to-face appointments. We suggest telephone assessment should be the first mode of communication with patients. Further studies should be conducted in other specialities to determine if there are similar outcomes.

## Introduction

The coronavirus disease 2019 (COVID-19) outbreak has caused major disruptions to healthcare services worldwide. With cases rising rapidly, elective operations were postponed or significantly reduced to minimize patient risks, release bed space, and allow surgeons and theatre staff to be redeployed to critical areas [1].

As of July 2021, there are an estimated 5.6 million patients awaiting elective operations, with 100,000 patients awaiting hip and knee joint replacement surgeries [2]. Postponing elective surgery will not only cause reduced mobility and increased pain but will also have a huge impact on patients’ overall wellbeing [3].

The World Health Organization defines harm as “temporary or permanent impairment of the physical, emotional, or psychological function or structure of the body and/or pain resulting there from requiring intervention” [4].

According to NHS England (NHSE), the definition of harm varies depending on the circumstances being investigated. In elective orthopaedics, harm is assessed by the 52-week Referral to Treatment Pathway (RTT) [5]. This is categorised as the following: none: no change in symptoms; low: prolongation of symptoms; medium: increase in symptoms and increase in medication or treatment; severe: irreversible progression of disease or death on the waiting list from index condition.

The aim of this study was to investigate the outcomes of a harm review service in hip and knee elective surgery arranged due to the postponement of their surgeries caused by the COVID-19 pandemic from March 2020 at a District General Hospital in East Kent NHS Foundation Trust. The primary objective of the study was to assess patient’s experiences of being on the waiting list for prolonged periods of time, specifically pertaining to the effect on their physical symptoms and mental state. The secondary objective was to assess the clinicians’ perspective, specifically whether alternative measures, for example, diagnosis, investigations, or procedures, were required for the patient.

## Materials and methods

All patients whose elective surgical procedures had been delayed for over 52 weeks due to the COVID-19 pandemic, were allocated to receive a ‘Harm Review’ consultation within the East Kent Hospitals NHS Trust.

Between April and June 2021, 242 patients whose elective orthopaedic (hip and knee) procedures had been sufficiently delayed at the William Harvey Hospital, Ashford, for more than 52 weeks due to the COVID-19 pandemic were offered a telephone harm review consultation by eight hip and knee orthopaedic consultants. Each consultant telephoned patients from their waiting lists, with each patient taking around 10-15 minutes to review, thus totalling around 60-80 hours of clinic time.

Patient demographic factors, diagnosis, and the procedure listed were recorded. With regards to the patients’ perspective, they were asked whether they perceived their physical symptoms and mental health status to have become better, worse, or unchanged. Furthermore, patients were asked whether they felt the ‘harm review’ telephonic consultation had been of value.

Regarding the clinicians’ perspective, the following were assessed: whether there was a change in clinical diagnosis; whether fresh investigations were required; whether further face-to-face consultation was necessary; whether patients were taken off their respective waiting lists; whether there was a change in the treatment plan; and, lastly, if there was significant harm done because of the increased waiting time for their elective hip or knee surgery. The reasons for any changes relating to the clinicians’ perspective mentioned above were also detailed.

We reviewed the data from 242 patients’ harm review consultations and then organised and tabulated the data in an Excel spreadsheet. An objective analysis was undertaken to ascertain the impact of the harm review telephonic service as it was a relatively new service whose impact was unknown. This research was registered and approved locally within the trust clinical audit department (registration number: RN707991).

## Results

**Figure 1:**
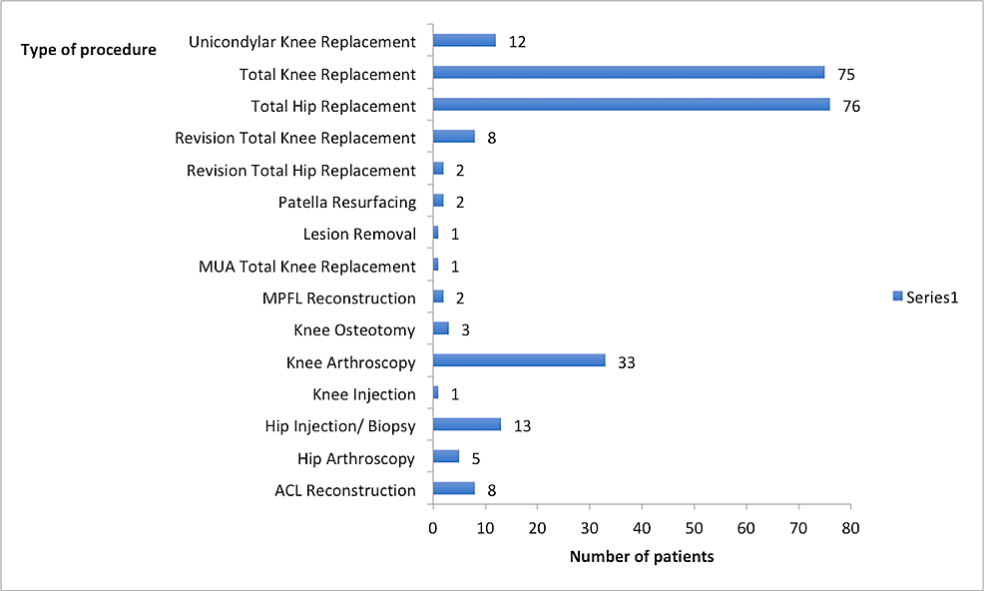
Elective procedures listed. MUA: manipulation under anaesthesia; MPFL: medial patellofemoral ligament; ACL: anterior cruciate ligament

There were a total of 242 patients who had a telephone consultation with eight lower limb orthopaedic consultants. In total, there were 127 females and 115 males. Of the 127 females, 49 had hip and 78 had knee problems. Of the 115 males, 46 had hip and 69 had knee problems. Figure 1 shows the types of operations the patients were listed for.

**Figure 2:**
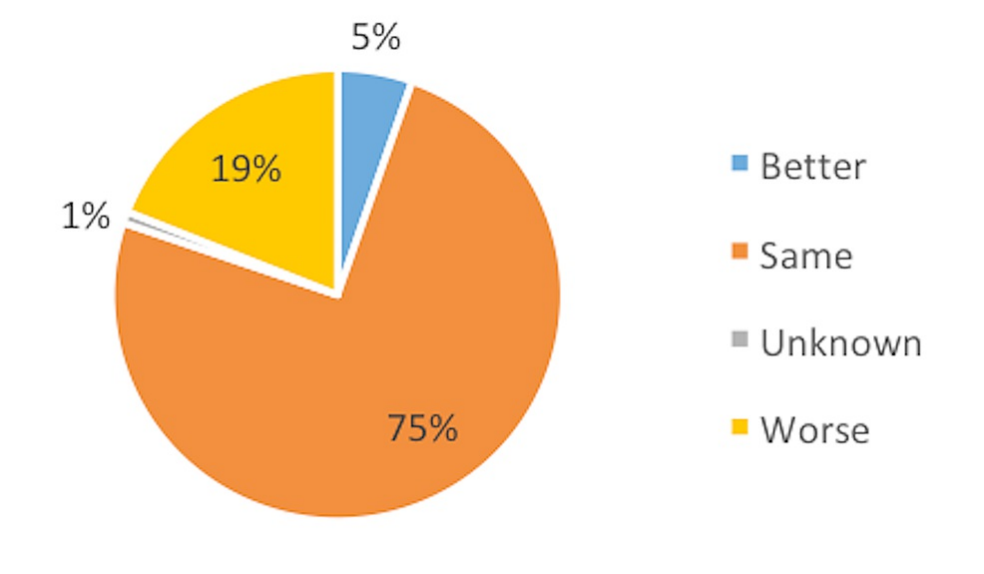
Patient symptoms.

Regarding symptoms, 182 (75%) patients felt the same. In total, 13 (5%) patients reported improvement in their physical symptoms, whereas 46 (19%) felt their symptoms were slightly or much worse (Figure 2).

**Figure 3:**
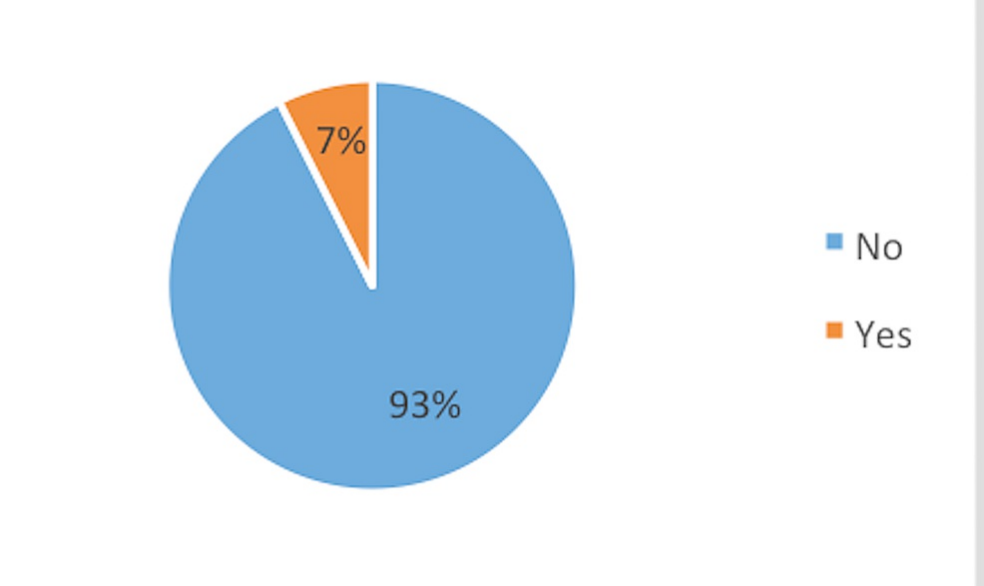
Face-to-face appointment.

Overall, 18 (7%) patients needed further face-to-face consultations for reassessment (Figure 3).

**Figure 4:**
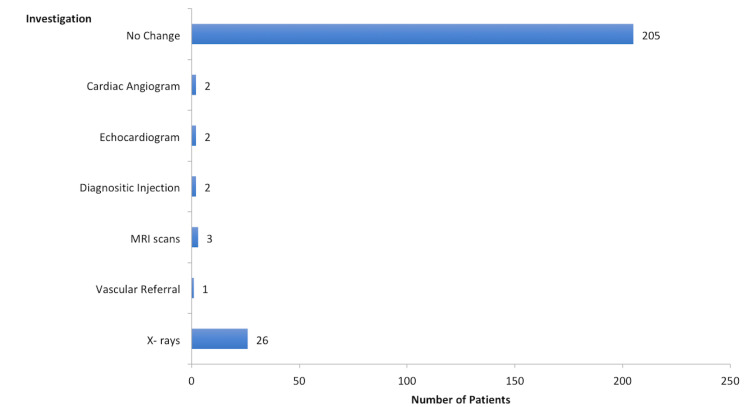
Further investigations required by the patients.

In total, 36 patients were sent for further investigations, as shown in Figure 4.

**Figure 5:**
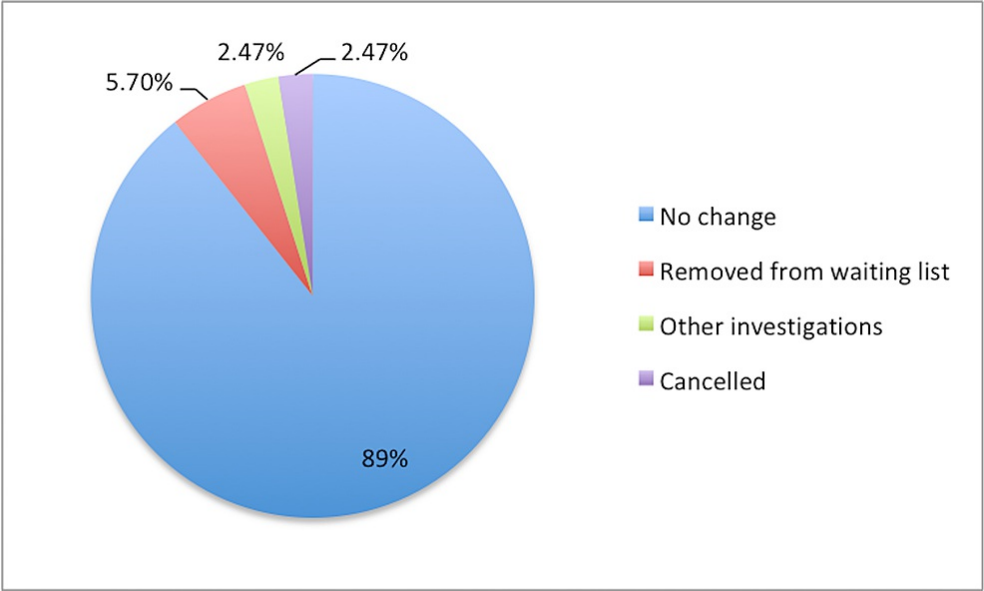
Changes to the treatment plan.

Overall, 26 (10.7%) patients had a change in their treatment plan following the consultation. Fourteen (5.7%) patients were taken off the waiting list as they got better or did not want an operation. Six (2.47%) patients had their operations cancelled due to other conditions (not related to the pandemic). Six (2.47%) patients were investigated for other problems or comorbidities, and their operation had to be postponed pending investigations (Figure 5).

**Figure 6:**
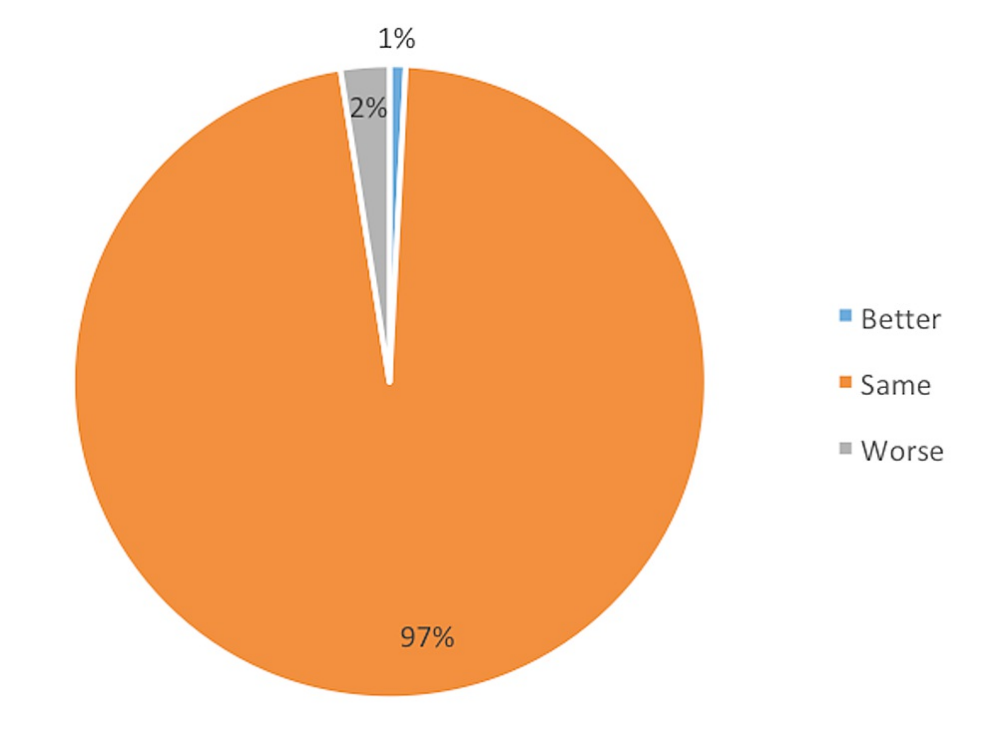
Mental status of the patients.

The majority of the patients (234 patients) felt there was no change in their mental status. Six (2%) patients mentioned that they were either frustrated, apprehensive, or anxious; however, none were depressed. Two (1%) patients reported that they felt mentally better as their physical symptoms improved (Figure 6).

**Figure 7:**
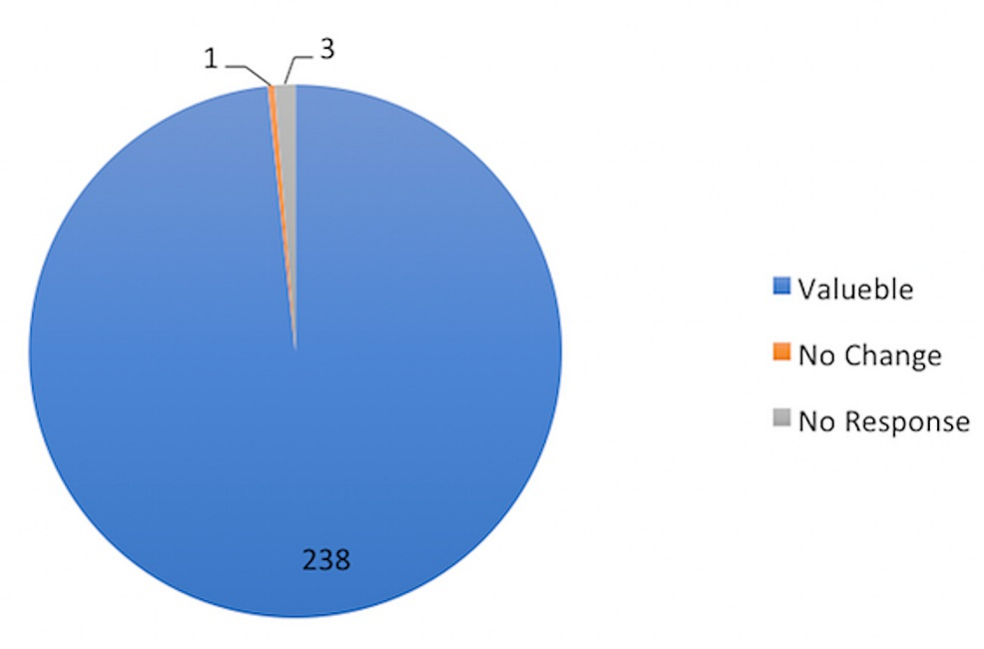
Value of consultation. Value: number of patients

In total, 238 (98%) patients felt the consultation was valuable and appreciated the service. However, three patients felt the consultation added no value, and one patient did not provide a response (Figure 7).

## Discussion

The COVID-19 pandemic has had a devastating impact on NHS services. Our results show that despite the postponement/cancellation and delay in surgery in 242 hip and knee elective surgeries, the majority of patients did not deteriorate in symptoms. In 46 (19%) patients, the pain became worse, whereas 13 (5%) patients felt their symptoms had improved.

Regarding mental health, the majority of the patients (96.7%) felt the same, whereas only 2.5% (six patients) felt worse. These results are surprising considering studies have shown that lockdown affected the mental health wellbeing of individuals [6,7].

Studies have reported a deterioration in the quality of life and symptoms in less than one-fifth of patients awaiting hip arthroplasty [8,9]. Similarly, another study reported a worsening of quality of life in about a quarter of patients awaiting knee arthroplasty, while another study showed progressive worsening of pain and disability after a wait time of more than a year [10,11]. Most studies were conducted by postal questionnaires rather than telephonic consultations which were unique since the COVID-19 pandemic [11].

Our study does not show the above-mentioned similar outcomes as these reviews were telephone consultations and happened during an unprecedented pandemic and activities were restricted. Hence, patients’ bodies and minds were diverted to the severity of the COVID-19 pandemic affection and caused an unusual change in attitude, behaviour, environment, and inactivity during the lockdown [12].

Studies have shown that telephone consultation results in high levels of patient satisfaction, implying that this modality of consultation could be useful in the future [13]. The majority of the patients (98%) found the consultation valuable and useful. Given that an average phone call may take 10-15 minutes, the total time translates to a minimum of 60 hours. Only 18 (7.4%) patients required a further face-to-face follow-up following the telephone consultation.

With an increasing number of COVID-19 cases and emerging variants, this pandemic might cause a further delay in elective surgery. We suggest that a pre-habilitation service can be incorporated [14]. This can include physical therapy rehab, virtual meditation, and Tai Chi, which can improve postoperative outcomes, especially for patients with osteoarthritis of the hips and knees [15,16].

## Conclusions

Our study demonstrated that the harm review service has been a valuable service to both patients and the orthopaedic department. This service was able to identify patients who no longer needed further treatment, thus reducing the waiting list time and saving resources. We feel that the telephone assessment of patients by medical staff should be a regular feature for patients whose operations get postponed beyond 52 weeks, especially in future pandemics and lockdowns. Regular telephonic harm review consultations are a better way of immediate assessment of patients rather than postal questionnaires or organising face-to-face clinic visits. Collation of multi-centric, multi-departmental audits of harm review clinics for other sub-specialities should be conducted to determine if there are similar outcomes.
